# Comparison of frequency and strain-rate domain mechanical characterization

**DOI:** 10.1038/s41598-018-31737-3

**Published:** 2018-09-12

**Authors:** Luca Bartolini, Davide Iannuzzi, Giorgio Mattei

**Affiliations:** 1 Biophotonics & Medical Imaging and LaserLaB, Vrije Universiteit Amsterdam, De Boelelaan 1105, 1081 HV Amsterdam, The Netherlands; 2 Optics11 B.V., De Boelelaan 1081, 1081 HV Amsterdam, The Netherlands; 3 Department of Anatomy and Embryology, Leiden University Medical Center, Einthovenweg 20, 2333 ZC Leiden, The Netherlands; 4 Department of Information Engineering, University of Pisa, Largo Lucio Lazzarino 1, 56122 Pisa, Italy

## Abstract

Indentation is becoming increasingly popular to test soft tissues and (bio)materials. Each material exhibits an unknown intrinsic “mechanical *behaviour*”. However, limited consensus on its “mechanical *properties*” (i.e. quantitative descriptors of mechanical behaviour) is generally present in the literature due to a number of factors, which include sample preparation, testing method and analysis model chosen. Viscoelastic characterisation – critical in applications subjected to dynamic loading conditions – can be performed in either the time- or frequency-domain. It is thus important to selectively investigate whether the testing domain affects the mechanical results or not. We recently presented an optomechanical indentation tool which enables both strain-rate (nano-$\dot{\varepsilon }M$) and frequency domain (DMA) measurements while keeping the sample under the same physical conditions and eliminating any other variability factor. In this study, a poly(dimethylsiloxane) sample was characterised with our system. The DMA data were inverted to the time-domain through integral transformations and then directly related to nano-$\dot{\varepsilon }M$ strain-rate dependent results, showing that, even though the data do not perfectly overlap, there is an excellent correlation between them. This approach indicates that one can convert an oscillatory measurement into a strain-rate one and still capture the trend of the “mechanical behaviour” of the sample investigated.

## Introduction

Indentation techniques at the micro- and nano-scale are becoming increasingly relevant in the mechanical characterization of materials, thanks to their numerous advantages such as relatively simple setups, ease of sample preparation, non-invasiveness and high-resolution^1^. While these techniques were initially proposed for metals and ceramic materials^2,3^, due to their ability of exerting small and confined loads, they developed into one of the primary tools to investigate soft polymers and biological tissues at typical cell length-scales^4–8^.

**Figure 1:**
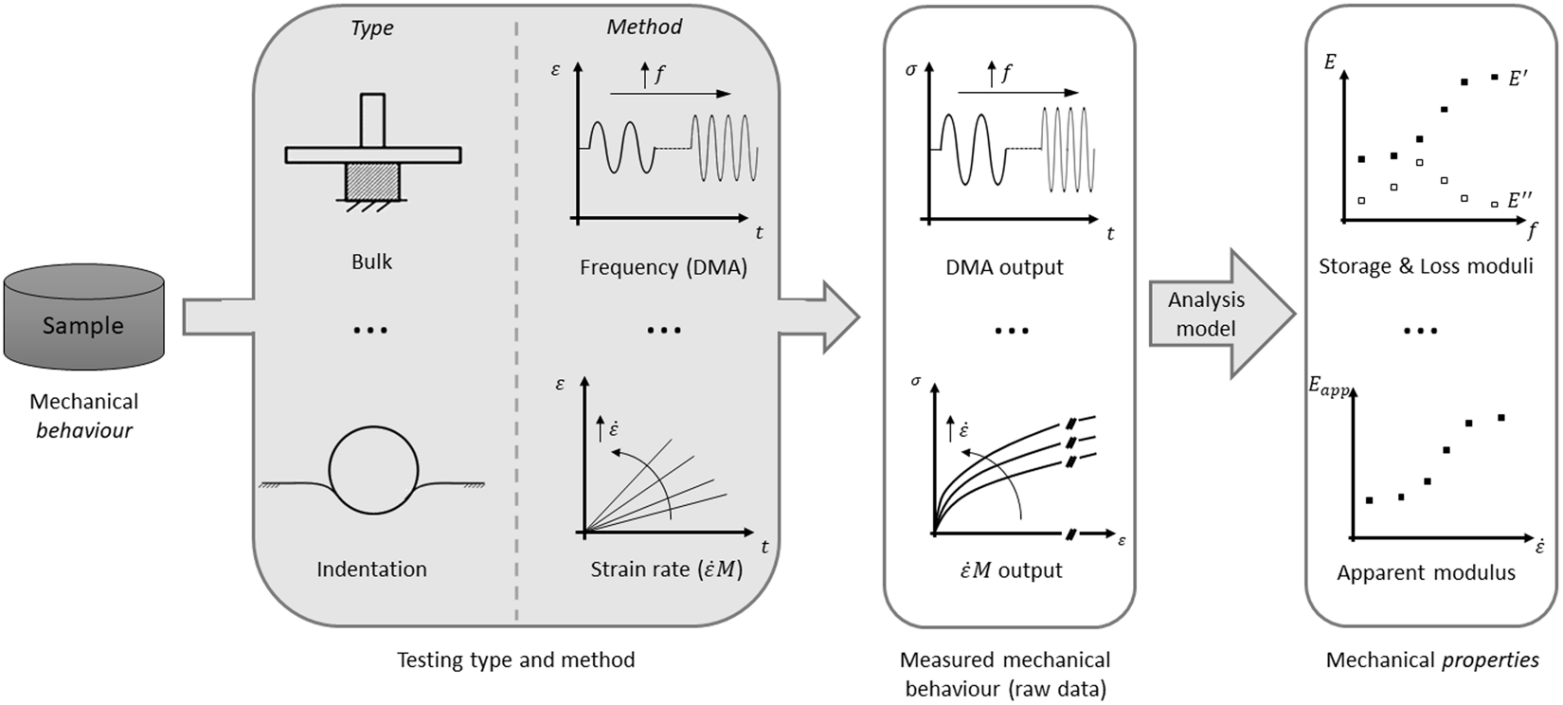
Distinction between sample mechanical *behaviour* and *properties*. Acronyms and symbols in figure: DMA = Dynamic Mechanical Analysis; $\dot{\varepsilon }M$ = epsilon dot method; *ε* = strain; $\dot{\varepsilon }$ = strain rate; *f* = frequency; *t* = time; *σ* = stress; *E* = elastic modulus; *E*′ = storage modulus; *E*″ = loss modulus; *E*_*app*_ = apparent elastic modulus.

It has already been discussed that every material comes with its specific, intrinsic “mechanical *behaviour*”, which cannot be known *a priori*^9^. The mechanical behaviour can be measured by means of different testing types and methods. Each measurement provides raw data sets that can be analysed with a given model to obtain specific “mechanical *properties*”, i.e. quantitative parameters describing the sample mechanical *behaviour* within the input range investigated (Fig. 1)^9^.

In general, viscoelastic characterization can be implemented either in the *time* (e.g. creep, stress relaxation, strain rate tests) or *frequency* domain (e.g. dynamic mechanical analysis), both at the macro- (e.g. bulk testing) and micro-scales (e.g. nano-indentation)^9^. While it is quite straightforward to compare results derived with the same testing method (e.g. DMA tests at different length-scales^10^), to relate data obtained in different testing domains (e.g. frequency and time) proves to be more challenging. Zeltmann *et al*. recently introduced an elegant approach to compare results obtained in the frequency and strain rate domain, and tested the method on data extracted from different sources^11^. The comparison of data obtained under different conditions, however, is not straightforward. The mechanical results, in fact, may depend on a number of testing and analysis variables, including boundary conditions of the specimen^12^, its preparation^13,14^, measurement length-scale^15–17^ and type of mechanical test^18,19^. Even changing the indenter geometry or size has been reported to considerably affect the testing results^20–23^. Moreover, the latter are likely dependent on the particular model used to analyse experimental data, because of different assumptions regarding, for instance, material anisotropies, non-linearities^24–26^, adhesion^27,28^, or time-dependency^19,29–31^.

In a recent series of papers, we have presented an optomechanical indentation tool^32^ that allows one to perform both strain-rate^33^ and frequency domain^10^ measurements while keeping the sample under the same physical conditions. To validate the model proposed by Zeltmann and his colleagues, and, therefore, reinforce the bridge between strain-rate and frequency domain measurements, we used our instrument to perform a series of frequency (Dynamic Mechanical Analysis, DMA^10^) and strain-rate (nano-epsilon dot method, nano-$\dot{\varepsilon }M$^33^) measurements on the very same PDMS sample, and, in this way, to selectively study the effect of the measurement domain, while excluding the influence of any other sample-, testing- or analysis-related variable^9^.

## Results

### Dynamic Mechanical Analysis (DMA)

**Figure 2:**
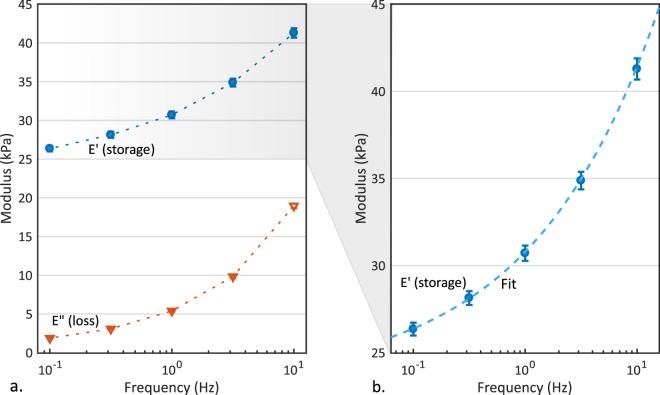
Dynamic mechanical analysis results obtained for PDMS. (**a**) Experimental storage (*E*′, blue circles) and loss (*E*″, orange triangles) moduli versus frequency (dotted lines represent linear data interpolation and only serve as a guide to the eye). (**b**) Storage modulus master curve (dashed line) obtained by fitting experimental *E*′(*f*) (blue circles) to the sigmoidal function $E^{\prime} (\omega )=a\cdot \,\tanh (b\cdot (\mathrm{ln}(\omega )+c))+d$, needed to derive time-domain results from DMA. Error bars denote standard errors.

Storage (*E*′) and loss (*E*″) moduli (Fig. 2a) were measured at 5 different logarithmically spaced frequencies (*f* = 0.100, 0.316, 1.00, 3.16, 10.0 Hz), performing *h*_0_ = 0.3 μm amplitude oscillations around a static *h*_*s*_ = 3 μm indentation depth^10^ (see Methods section for details).

A storage modulus master curve was derived by fitting experimental *E*′(*f*) data to a sigmoidal function (Eq. 10, Methods). Notably, this function is not intended to represent a specific viscoelastic model, but rather it was used here only as a mean to extrapolate a physically-relevant storage-modulus master-curve from experimental data. That master-curve was then used to compute the time-domain relaxation function, necessary to compare frequency (DMA) and strain-rate (nano-$\dot{\varepsilon }M$) domain results as described in the Methods section. Fitting results were: *a* = 196.9; *b* = 0.2006; *c* = −11.74; *d* = 220.4 (Fig. 2b).

### Nano-epsilon dot method (nano-$\dot{{\boldsymbol{\varepsilon }}}{\boldsymbol{M}}$)

**Figure 3:**
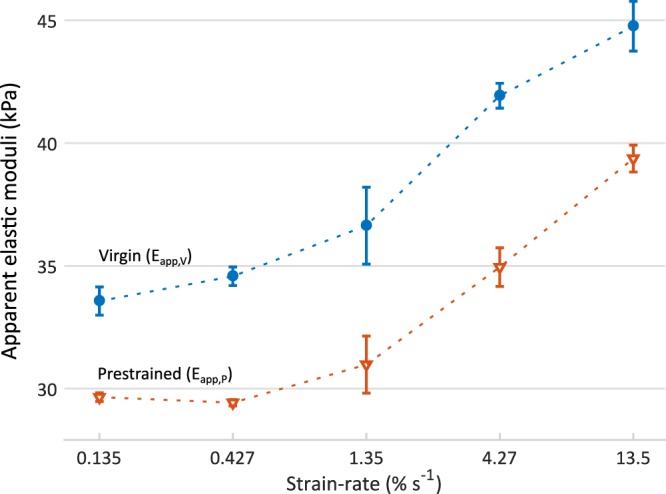
Strain-rate dependent apparent elastic moduli obtained for *virgin* (*E*_*app*,*V*_, blue circles) and *pre-strained* (*E*_*app*,*P*_, orange triangles) PDMS. Dashed lines are linear interpolation and only serve as a guide to the eye. Error bars denote standard errors.

Nano-epsilon dot measurements were performed at 5 different logarithmically spaced strain-rates ($\dot{\varepsilon }$ = 0.00135, 0.00427, 0.0135, 0.0427, 0.135 s^−1^). The nano-$\dot{\varepsilon }M$ has been originally developed to derive *virgin material properties*, i.e. in absence of any pre-strain/stress. However, nano-$\dot{\varepsilon }M$measurements were started at the same level of *static pre-strain* required by DMA (i.e. *h*_*s*_ = 3 μm), to obtain meaningfully comparable results between the two methods. Measurements were also performed in absence of pre-strain, to relate *virgin* and *pre-strained* apparent elastic moduli: ${E}_{app,V}(\dot{\varepsilon })$ and ${E}_{app,P}(\dot{\varepsilon })$, respectively. The linear viscoelastic region (LVR) extended up to 0.0175 strain in both sample conditions with strain-rate-increasing moduli (as expected for viscoelastic materials) exhibiting similar trends (Fig. 3).

### Comparing frequency and strain-rate domain results

The storage modulus master curve obtained fitting experimental *E*′(*f*) data from DMA was integrated numerically according to Eq. 11 (Methods) to derive the time-domain relaxation-modulus function, *E*(*t*), needed to compare frequency-domain (DMA) and strain-rate domain (nano-$\dot{\varepsilon }M$) results. Since *E*(*t*) ≥ 0, ∂*E*(*t*)/∂*t* ≤ 0 and ∂^2^*E*(*t*)/∂*t*^2^ ≤ 0, the computed *E*(*t*) solution satisfies the requirements of fading memory and non-negative stored and dissipated energy in the whole time range 0 to ∞^34,35^. The *E*(*t*) was then integrated according to Eq. 13 to obtain the stress-time responses to the constant strain-rate inputs of interest (i.e. the same used for nano-$\dot{\varepsilon }M$ measurements, namely $\dot{\varepsilon }$ = 0.00135, 0.00427, 0.0135, 0.0427, 0.135 s^−1^). The integral was solved numerically up to *t* = 13 s with 2 ms time steps, to have enough data and resolution to derive the stress-strain response within the LVR strain range (*ε* = 0–0.0175). Indeed, computing the stress-strain response within LVR for the lowest strain-rate of interest requires solving up to a time ${t}_{LVR}={\varepsilon }_{max}/{\dot{\varepsilon }}_{min}=0.0175/0.00135\approx 12.96\,{\rm{s}}$, while for the highest strain-rate solving up to ~ 0.13 s is required. Eventually, the DMA-derived apparent elastic moduli, ${E}_{app,DMA}(\dot{\varepsilon })$, were computed as the stress-strain slope within the same LVR used for nano-$\dot{\varepsilon }M$ characterisation, and compared to the ${E}_{app,P}(\dot{\varepsilon })$ resulting from pre-strained nano-$\dot{\varepsilon }M$ measurements (Fig. 3). This framework allowed us to meaningfully compare strain-rate and frequency derived mechanical results, and to investigate the specific effect of the testing domain.

**Figure 4:**
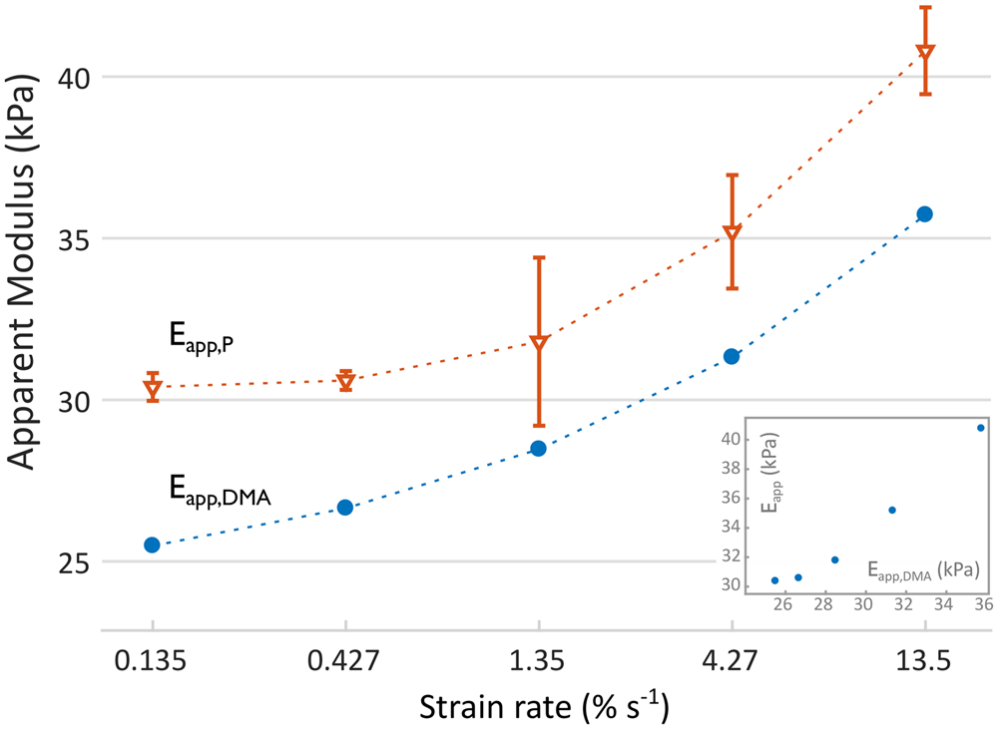
Strain-rate dependent apparent elastic moduli at the same level of pre-strain (i.e. 3 μm indentation depth), as obtained by nano-$\dot{\varepsilon }M$ (*E*_*app*,*P*_, orange triangles) and derived from DMA (*E*_*app*,*DMA*_, blue circles). Dashed lines are linear interpolation and only serve as a guide to the eye. Error bars denote standard errors. The inset shows the correlation plot between ${E}_{app,DMA}(\dot{\varepsilon })$ and ${E}_{app,P}(\dot{\varepsilon })$ data (*r* = 0.99).

Figure 4 shows an increase of *E*_*app*,*DMA*_ with strain-rate, as expected for viscoelastic materials and in agreement with experimental ${E}_{app,P}(\dot{\varepsilon })$ results. Overall, the ${E}_{app,P}(\dot{\varepsilon })$ values were higher than their respective ${E}_{app,DMA}(\dot{\varepsilon })$ ones, regardless of the strain-rate. However, an optimal correlation was observed between ${E}_{app,DMA}(\dot{\varepsilon })$ and ${E}_{app,P}(\dot{\varepsilon })$ data (*r* = 0.99), with an almost constant difference in the apparent moduli, regardless of the strain-rate.

## Discussion

The investigation of the testing domain effect on the ensuing mechanical results is of primary interest in material engineering and design^36^. Despite the large amount of DMA data available in the literature for several materials, frequency-domain results obtained with this method have rarely been used to design structures and components, mainly because they are not directly applicable to most of engineering problems. Conversely, mechanical properties derived in the strain-rate domain will be more convenient in such applications, but they are generally more challenging to obtain mostly because of limited speed ranges attainable with a given testing setup, and the quite long experimental trials needed to perform measurements at very low strain-rates, which result in time consuming and limited throughput tests^11^.

Frequency-dependent storage (*E*′) and loss (*E*″) moduli were obtained from DMA measurements at 5 different log-spaced frequencies (*f* = 0.100, 0.316, 1.00, 3.16, 10.0 Hz) on PDMS samples. In particular, both *E*′ and *E*″ increased with frequency, consistently with previous DMA results obtained at micro- and macro-scale^10,37^. In the assumption of simple rheological behaviour with one characteristic relaxation frequency (or time) for PDMS^33,38–40^, one could expect a peak in the *E*″ spectrum in correspondence of that frequency and a correspondent smooth transition in *E*′ spectrum, from the asymptotes of i) *equilibrium* (or relaxed) modulus at low frequencies, to ii) *instantaneous* modulus at high frequencies^19^. Since *E*′ and *E*″ show no asymptotic plateau and *E*″ keeps increasing with frequency, the material relaxation frequency is expected to be outside and to the right of the 0.1–10 Hz range investigated, in agreement with previous works^10,37^.

Nano-$\dot{\varepsilon }M$ measurements were performed at 5 different log-spaced strain-rates ($\dot{\varepsilon }$ = 0.00135, 0.00427, 0.0135, 0.0427, 0.135 s^−1^). Notably, nano-$\dot{\varepsilon }M$ measurements started at the same level of static pre-strain needed by DMA to perform dynamic oscillations (i.e. 3 μm indentation depth in this study), in order to measure the sample in the same fully-relaxed pre-strained equilibrium state and rule-out any depth-related nonlinearity^9,19,41,42^. Since the nano-$\dot{\varepsilon }M$ does not require any static pre-strain to start measurements^33^, it was also employed to derive *virgin* (i.e. not pre-strained) properties. Both the *virgin* (*E*_*app*,*V*_) and *pre-strained* (*E*_*app*,*P*_) apparent elastic moduli exhibited a similar increasing trend with strain-rate ($\dot{\varepsilon }$), as expected for PDMS at micro- and macro-scales^33,40,43^. A simple rheological behaviour with one characteristic relaxation time (as assumed for DMA) results in an apparent elastic modulus increasing with strain-rate, from an asymptotic *equilibrium* value at low strain-rates to a higher *instantaneous* one^44,45^. While a slow increase in both *E*_*app*,*V*_ and *E*_*app*,*P*_ was observed at low strain-rates, suggesting that these moduli are approaching the respective equilibrium values expected for $\dot{\varepsilon }\to 0$, no upper plateau values were observed with increasing strain-rates, suggesting that the transition to the *instantaneous* modulus would be completed beyond the highest strain-rate investigated (0.135 s^−1^), in agreement with other reports^33,40^. Notably, the ${E}_{app,V}(\dot{\varepsilon })$ values were higher than ${E}_{app,P}(\dot{\varepsilon })$ ones, regardless of the strain-rate. This result is consistent with Charitidis, reporting on the existence of a PDMS surface/near surface region (extending up to ~2.5 μm indentation depth) characterized by higher elastic moduli that significantly decrease with penetration depth and plateau to a lower *bulk* value^41^.

The *closed-loop* (feedback) control scheme adopted in this work enables constant indentation rate ($\dot{h}$) measurements, which represents a notable advancement with respect to the *open loop* control (D-mode) employed in previous nano-$\dot{\varepsilon }M$ tests^33^. Indeed, although in a D-mode controlled measurement a constant piezo displacement rate results in a nearly constant strain-rate ($\dot{\varepsilon }$) within the region of small deformations, the actual $\dot{\varepsilon }$ experienced by the sample is generally decreasing over time, and lower than the value calculated by imposing $\dot{h}$ equal to the piezo displacement rate in Eq. 7 ^7,33^. In particular, in a constant piezo displacement rate experiment, the higher the indentation depth into the sample surface, the higher the cantilever deflection rate because of the increased contact area between its spherical tip and the sample, which, in turns, results in a lower actual indentation rate (and strain-rate) experienced by the sample as the tip advances during measurements^7,33^. The closed-loop control scheme solves this problem, by adapting the piezo displacement rate during measurements in order to enable constant indentation rate experiments (I-mode), resulting in a constant $\dot{\varepsilon }$ experienced by the sample over the entire indentation range. This is also critical to perform nano-$\dot{\varepsilon }M$ measurements at a given level of pre-strain, required, for instance, to investigate the strain-dependency of mechanical properties and to compare nano-$\dot{\varepsilon }M$ and DMA results, as presented in this work.

We obtained apparent elastic moduli at different strain-rates in two ways: directly from nano-$\dot{\varepsilon }M$ measurements, ${E}_{app,P}(\dot{\varepsilon })$, and indirectly after integration of DMA data, ${E}_{app,DMA}(\dot{\varepsilon })$. We found an excellent correlation between them (r = 0.99, Fig. 4), with a constant undershoot of ${E}_{app,DMA}(\dot{\varepsilon })$ with respect to ${E}_{app,P}(\dot{\varepsilon })$. The average deviation between *E*_*app*,*P*_ and *E*_*app*,*DMA*_, equal to −10.26 ± 1.02% (mean ± std. err.), is consistent with that reported by Zeltmann *et al*.^11,36^, and indicates an overall good agreement between frequency and strain-rate domain results. Such discrepancy indicates the presence of a systematic error, possibly owed to the narrow frequency range attainable by our testing setup. Notably, outside of that 0.1–10 Hz range, the accuracy of the time-domain relaxation function depends on the extrapolation of the fitted storage-modulus master function. Possible *E*′ transitions (and concomitant *E*″ peaks) outside the measured frequency range are indeed not considered and may cause deviations from the true relaxation function of the material under testing. Given the maximum frequency of 10 Hz investigated in this work, the smallest time from which the relaxation function reflects experimental data can be estimated as *t* → 1/*f* ^35^, corresponding to a lower limit of 0.1 s; at shorter times, the relaxation function is primarily influenced by the extrapolation of the storage modulus fit at frequencies >10 Hz. At the maximum strain-rate investigated in this work, the upper limit of the LVR is reached in ${t}^{\dagger }=\,{\varepsilon }_{max}/{\dot{\varepsilon }}_{max}=0.0175/0.135\approx 0.13$ s, meaning that the respective *E*_*app*,*DMA*_ value is largely derived from extrapolated data (though all times are affected to some extent by all frequencies, and vice versa). However, since the difference between *E*_*app*,*P*_ and *E*_*app*,*DMA*_ values was almost independent of the strain-rate, there is good evidence for a systematic error in the evaluation of DMA moduli. We investigated this possibility by a variation analysis on the parameters of the fit. In particular, a 2% increase in the parameter *d* (i.e. the offset addend in Eq. 10) results in an almost perfect match between *E*_*app*,*P*_ and *E*_*app*,*DMA*_, and a small upward transition of the master curve. If systematic error on DMA had the same magnitude, there would be no discrepancy between *E*_*app*,*P*_ and *E*_*app*,*DMA*_.

## Conclusion

In this work we show a direct comparison between mechanical results derived from strain-rate and frequency domain measurements, in a setting where – for the first time – any other sample-, testing- and/or analysis-related source of variability was eliminated^9^. The demonstration that testing in either the frequency or strain-rate domain returns compatible results is critical towards a more comprehensive understanding of material viscoelastic (i.e. time-dependent) behaviour, which is of primary importance for a number of applications, ranging from mechanical and structural design for civil and (bio)material engineering^46,47^, to tissue engineering^48,49^ and cell mechano-biology^50,51^.

## Methods

### Sample preparation

Polydimethylsiloxane (PDMS) samples with flat surfaces were obtained by casting a Sylgard 184 (Dow Corning) pre-polymer solution (50:1 curing agent to elastomer weight ratio, prepared as per manufacturer’s instructions) into a 50 mm diameter Petri dish. The solution was degassed for 15 minutes and cured overnight at 60 °C. The surface of the sample was then passivated with a 5% bovine serum albumin solution (BSA, Sigma Aldrich) in order to minimize adhesion forces^10^, which invalidate the Hertzian contact assumption of our analyses. Indentation measurements were carried out in phosphate buffered saline (PBS 1x, further reducing tip-sample adhesion^52^) at a controlled temperature of 25 °C. The Poisson’s ratio of PDMS was assumed to be *ν* = 0.5^10,33,53^.

### Nano-indentation setup

Indentation experiments were performed with a custom setup based on an opto-mechanical ferrule-top cantilever sensor^10,54^, equipped with an *R* = 248 μm radius spherical tip, measured with a microscope. A commercial interferometer (OP1550, Optics11 B.V.) was used to measure the deflection of the cantilever via Fabri-Perót interferometry^54^.

**Figure 5:**
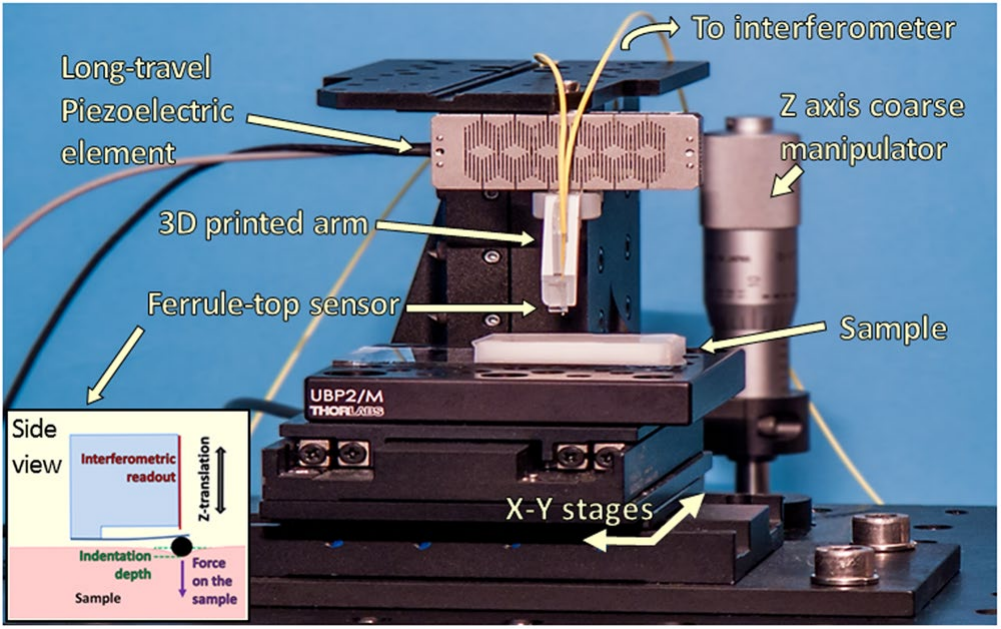
Nano-indentation setup (adapted from our previous work on multimodal ferrule-top sensing^8^). The inset shows a schematic side view of our ferrule-top cantilever sensor operated by a vertical piezoelectric actuator. The load imparted on the sample surface by the spherical tip is equal to the cantilever stiffness times its deflection (read via interferometry), while the resultant indentation depth into the sample is given by the difference in between piezo-motor displacement downwards and the resultant cantilever deflection upwards.

The experimental setup is shown in Fig. 5. A custom 3D-printed rigid arm was used to mount the probe on a long-range piezoelectric actuator (PI-602.3SL, 300 μm range, Physik Instrumente GmbH), which, in turn, was attached to a manual positioning z-stage manipulator with a 12.5 mm travel length (MVS005, Thorlabs) to coarsely approach the sample before performing the piezo-controlled indentation experiment. The sample was placed on a XY motorized stage actuated by 2 orthogonal linear stages (M664.164 stages, C867 controllers, Physik Instrumente GmbH), which allowed for XY sample positioning and automated surface scanning with an accuracy of less than 1 μm.

Indentation experiments were performed at controlled temperature, by enclosing the whole setup in a custom wooden isolation box lined with phono-absorbent foam, which provides both acoustic and thermal insulation. The temperature inside the box was measured by a PT100 Platinum Resistance Thermometer (Labfacility DM-508, Farnell), connected to a PID controller (Eurotherm 94, ELEKTRO-TRADING) which drove the heating elements (6 × 1 Ohm high-power resistors) to maintain a constant temperature during measurements.

The stiffness of the ferrule-top cantilever (*k*_*c*_) was determined following a calibration method (assuming a linear spring behaviour)^54^, which resulted in *k*_*c*_ = 8.2 N/m. Given the tip radius (i.e. 248 micron) and the range of sample elastic moduli (~25–40 kPa), this cantilever stiffness allows to obtain a measurable cantilever bending with good signal-to-noise ratio (thus measurable load) and enough indentation into the sample surface. For instance, testing extremely soft materials with stiff cantilevers would result in a negligible cantilever bending, turning all the piezo movement downwards (*d*_*p*_) into sample indentation (*h*) and preventing to measure the resultant load via cantilever deflection (*d*_*c*_): please refer to Eqs 1 and 2 in the following section. Conversely, testing extremely hard materials with very soft cantilever would result in *d*_*c*_ almost equal to *d*_*p*_, with negligible sample indentation (*h*). Before each experiment, probe performances were tested by a calibration procedure on glass in the same conditions used to test samples (i.e. in PBS 1x at 25 °C)^55^.

### Displacement-, load- and indentation-control mode

Two physical quantities can be measured with the presented setup: (i) the load imparted from the spherical tip to the sample surface, and (ii) the resultant indentation depth into the sample. According to Hooke’s law, the load (*P*) can be derived as the product of the cantilever stiffness (*k*_*c*_) times its deflection (*d*_*c*_) as follows:

1 $$P={k}_{c}\cdot {d}_{c}$$

After establishing contact with the sample surface, the indentation depth (*h*) is given by the difference between the piezo displacement (*d*_*p*_) and the resultant cantilever deflection (*d*_*c*_):

2 $$h={d}_{p}-{d}_{c}$$

The simplest operation mode of our setup is the displacement-control (D-mode), in which we prescribe a given piezo displacement time-profile *d*_*p*_(*t*), and measure the resultant *P*(*t*) and *h*(*t*). Notably, D-mode (controlling only the piezo displacement regardless of the cantilever deflection) does not allow a direct control of any of the latter two variables, which is instead needed for dynamic mechanical analysis^10^ or nano-epsilon dot tests^33^. To enable load (P-mode) or indentation (I-mode) controlled measurements, we have developed a *closed-loop* (feedback) control scheme, which reads the cantilever deflection and adjusts the piezo displacement at 500 Hz refresh rate. An *on-the-fly* determination of the contact point between the indenter tip and the sample surface was implemented to trigger (i.e. switch on) the closed loop control. Notably, the activation of the feedback loop when out-of-contact results in a divergence of the input signal, jerking the probe into the sample and inevitably breaking the cantilever. In particular, the cantilever deflection was monitored in real-time after starting nano-indentation measurements out of sample contact, and the latter was identified as the instant at which the cantilever bending was higher than 2.5 times the standard deviation of the interferometric readout measured out of sample contact (i.e. instrumental noise). This threshold guaranteed good contact point identification, with negligible onset delay for either the P- or I-mode feedback loop and irrelevant sample pre-stress. Data readout and instrument control were performed with a custom LabView (National Instruments) software.

### Dynamic mechanical analysis (DMA)

Dynamic mechanical analysis is a technique for material viscoelastic characterization in the frequency domain, which has been applied also to nano-indentation^10,21,55^. Briefly, DMA is based on the application of a cyclic (sinusoidal) input of displacement or load with frequency *f* and amplitude *h*_0_ or *P*_0_, respectively, and the measurement of the resultant load or displacement response. If the amplitude of the input oscillation is small enough, the measurements occur within the material Linear Viscoelastic Region (LVR), therefore the stress response is expected to be i) independent of the input amplitude, ii) sinusoidal with the same frequency as the input (*f*), and iii) present a phase lag *ϕ*. The latter is 0° for a purely elastic material, 90° for a purely viscous material, and in-between 0° and 90° for any viscoelastic material^56^.

**Figure 6:**
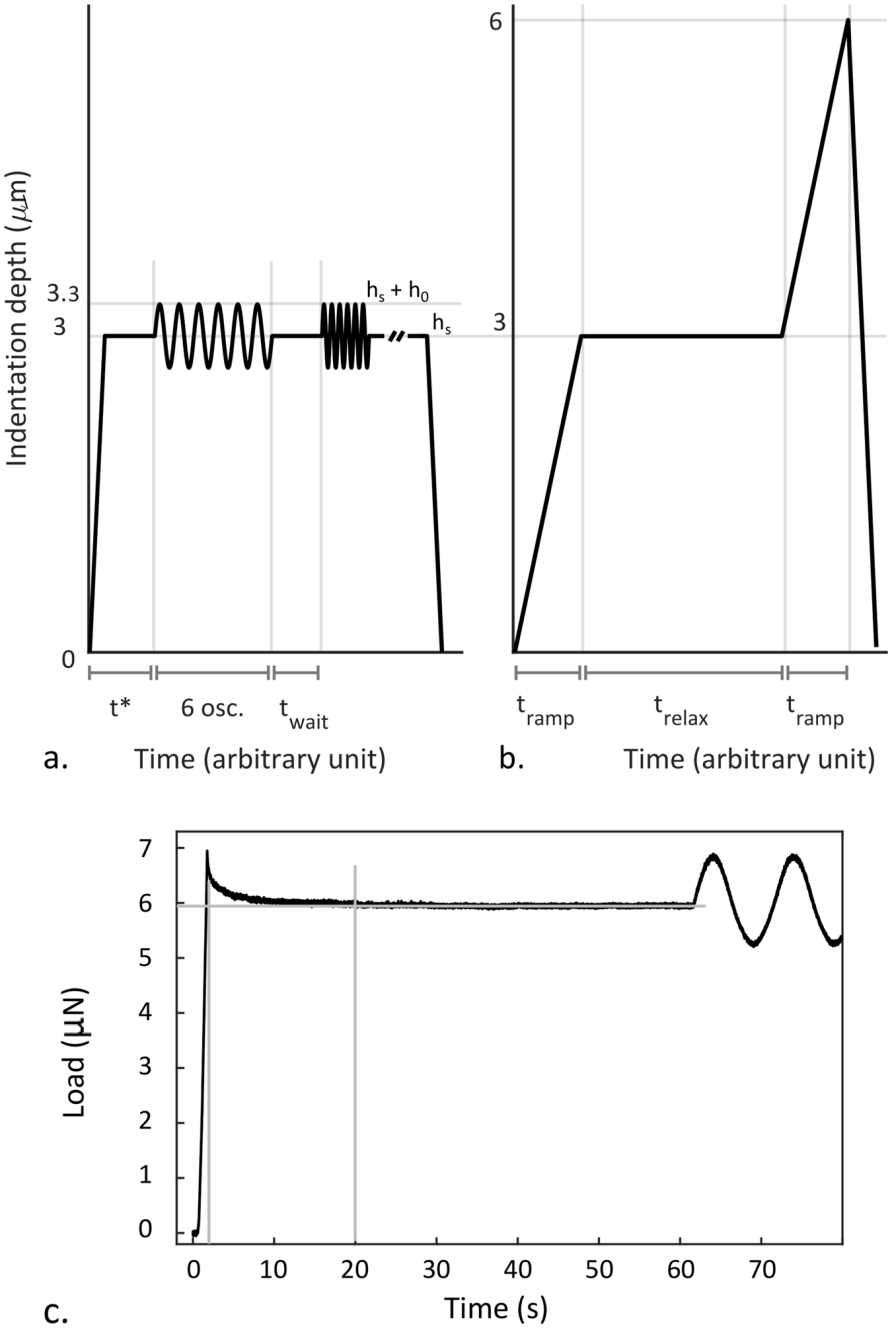
Prescribed indentation profiles for (**a**) DMA and (**b**) nano-$\dot{\varepsilon }M$ measurements. (**c**) Experimental data showing that the load plateau after ramping to *h*_*s*_ = 3 μm was reached in less than 20 s, as highlighted by the light grey lines.

In the present study, indentation-controlled (I-mode) DMA experiments were carried out, performing *h*_0_ = 0.30 μm dynamic oscillations around a static *h*_*s*_ = 3 μm indentation depth. These testing parameters satisfy the requirement of small indentation depth with respect to tip radius, offer a good *signal-to-noise* ratio of the cantilever deflection, eliminate the risk of tip-sample detachment during oscillations, and guarantee the measurement is within the LVR of PDMS^10,21^. The DMA oscillations were carried out at 5 logarithmically spaced frequencies (*f* = 0.100, 0.316, 1.00, 3.16, 10.0 Hz). Measurements started out of contact: after an *on-the-fly* contact identification, the I-mode controlled piezoelectric actuator moved the probe downwards to the static indentation depth *h*_*s*_ = 3 μm in *t*_*ramp*,*DMA*_ = 1 s, which was then held constant for *t*_*relax*_ = 60 s, allowing the sample to reach a new fully relaxed pre-strained equilibrium state (as shown in Fig. 6c) around which to perform DMA oscillations (starting at t* = *t*_*ramp*,*DMA*_ + *t*_*relax*_ = 61 s in the schematic shown in Fig. 6a)^55^.

Tests at different frequencies were carried out consecutively (with *t*_*wait*_ = 2 s waiting time in between them, Fig. 6a), by performing 6 oscillations of the indentation depth and measuring the resultant sinusoidal load response.

Experimental indentation and load data obtained at each frequency were fitted to a sinusoid, in order to derive *h*_0_, *P*_0_ and, which were then used to compute the storage, *E*′(*f*), and loss, *E*″(*f*), moduli, according to the following equations^21^.

3 $$\frac{E^{\prime} }{(1-{\nu }^{2})}=\frac{{P}_{0}}{{h}_{0}}\,\cos (\varphi )\frac{1}{2\sqrt{{h}_{s}R}}$$

4 $$\frac{E^{\prime\prime} }{(1-{\nu }^{2})}=\frac{{P}_{0}}{{h}_{0}}\,\sin (\varphi )\frac{1}{2\sqrt{{h}_{s}R}}$$

DMA measurements were performed on *n* = 36 different (randomly selected) surface points spaced at least by 500 µm, in order to avoid any possible effect from repeated testing cycles on the same spot^7,33^. Each measurement comprises all the 5 frequencies of interest, thus resulting in *n* = 36 replicates per frequency investigated. A custom MATLAB (The Mathworks Inc.) code was used to process the data.

### Nano-epsilon dot method (nano-$\dot{{\boldsymbol{\varepsilon }}}{\boldsymbol{M}}$)

The nano-epsilon dot method (nano-$\dot{\varepsilon }M$) stems from the epsilon dot method ($\dot{\varepsilon }M$), a technique for material viscoelastic characterization in the strain-rate domain^19^. The $\dot{\varepsilon }M$ is based on performing mechanical tests at different constant strain-rates, reporting a strain-rate dependent *apparent* elastic modulus, ${E}_{app}(\dot{\varepsilon })$. The latter is defined as the slope of the stress-strain response of the material within the LVR and increases with strain-rate ($\dot{\varepsilon }$) in case of viscoelastic materials. The $\dot{\varepsilon }M$ has been recently adapted from bulk testing to nano-indentation, to obtain the nano-$\dot{\varepsilon }M$, which introduces a new definition of indentation stress (*σ*) and strain (*ε*) based on the Hertzian contact^33^.

5 $$\sigma =\frac{P}{R\sqrt{hR}}$$

6 $$\varepsilon =\frac{4}{3(1-{\nu }^{2})}(\frac{h}{R})$$

Thanks to the new definitions (Eqs 5 and 6), the indentation strain-rate ($\dot{\varepsilon }=\partial \varepsilon /\partial t$) is independent of the indentation strain (*ε*) and linearly proportional to the indentation rate ($\dot{h}$) as follows:

7 $$\dot{\varepsilon }=\frac{\partial \varepsilon }{\partial t}=\frac{4}{3(1-{\upsilon }^{2})}(\frac{\dot{h}}{R})$$

Therefore, to implement a constant strain-rate test at a desired ${\dot{\varepsilon }}_{ind}$, one has simply to impose a constant indentation rate ($\dot{h}$), obtained by reverting Eq. 7.

In this work, tests were performed at 5 logarithmically spaced strain-rates chosen as representative of the 5 logarithmically spaced frequencies used for DMA experiments (0.100–10.0 Hz). Notably, the conversion of those frequencies to a constant strain-rate is inherently impossible, because the strain-rate is continuously varying during oscillations at a constant frequency. Indeed, the sinusoidal oscillation of the indentation input results in a cosinusoidally varying strain-rate. To choose 5 representative constant strain-rates for the nano-$\dot{\varepsilon }M$, we calculated the instantaneous strain-rate in the quasi-linear portion at the beginning of the oscillation, i.e. the first time derivative of the oscillating strain across the static indentation depth *h*_*s*_ = 3 μm around which oscillations with amplitude *h*_0_ = 0.30 μm and frequency *f* are performed. Considering the indentation input equal to $h={h}_{s}+{h}_{0}\,\sin (2\pi ft)$, the strain rate (defined as in Eq. 7) is given by

8 $$\dot{\varepsilon }=\frac{4}{3R(1-{\upsilon }^{2})}\frac{\partial h}{\partial t}=\frac{4}{3R(1-{\upsilon }^{2})}2\pi f\,{h}_{0}\,\cos (2\pi ft)$$

Assuming $\cos (2\pi ft)=1$ in the quasi-linear portion around the static oscillation dept*h h*_*s*_, the former equation simplifies to:

9 $$\dot{\varepsilon }=\frac{4}{3R(1-{\upsilon }^{2})}2\pi f{h}_{0}\approx 11.18\cdot \frac{f\cdot {h}_{0}}{R}$$

from which representative strain-rates for nano-epsilon dot measurements were derived (by substituting *h*_0_ = 0.30 μm; *R* = 248*μm*; f = 0.100, 0.316, 1.00, 3.16, 10.0 Hz), obtaining $\dot{\varepsilon }$ = 0.00135, 0.00427, 0.0135, 0.0427, 0.135 s^−1^.

Nano-$\dot{\varepsilon }M$ measurements started out of contact. After finding the contact point *on-the-fly*, the I-mode controlled piezoelectric actuator moved the probe downwards at the desired indentation rate $\dot{h}$ up to *h*_*s*_ = 3 μm depth (i.e. the static indentation depth value used in DMA experiments; *t*_*ramp*_ in Fig. 6b). Consistently with the DMA testing protocol, this indentation depth was held constant for *t*_*relax*_ = 60 s (Fig. 6b), to allow the sample to reach the same, fully relaxed, pre-strained, equilibrium state as in DMA measurements. The relaxed and pre-strained material was then probed with a second constant strain-rate test at the same $\dot{h}$ (thus $\dot{\varepsilon }$) as the first ramp, to obtain pre-strained apparent elastic moduli, that are meaningfully comparable with those derived from DMA measurements.

Experimental load-indentation (*P* − *h*) data were converted into stress and strain according to Eqs 5 and 6, respectively. Data belonging to the first ramp were used to derive the strain-rate dependent apparent elastic moduli for the *virgin* material (${E}_{app,V}(\dot{\varepsilon })$, obtained in absence of pre-strain) as the slope of the first linear tract of the *σ* − *ε* curve^7,33^, which identifies the *absolute* linear viscoelastic region (LVR)^19^. Strain-rate dependent elastic moduli for *pre-strained* material, ${E}_{app,P}(\dot{\varepsilon })$, were derived in the same manner from data belonging to the second ramp, i.e. within a *local* LVR. Notably, the measurement on the pre-strained material started at the same level of pre-strain necessary for DMA experiments (*h*_*s*_ = 3), and in the same equilibrium relaxed state. The ${E}_{app,P}(\dot{\varepsilon })$ data can thus be meaningfully compared with DMA results^9,19^.

Nano-$\dot{\varepsilon }M$ measurements were performed on *n* = 30 different (randomly selected) surface points spaced at least by 500 µm, obtaining *n* = 6 replicates for each of the 5 strain-rates investigated. Data were processed with a custom MATLAB (The Mathworks Inc.) code.

### Comparing DMA and nano-$\dot{{\boldsymbol{\varepsilon }}}{\boldsymbol{M}}$ results

To compare results obtained from frequency-domain (DMA) and strain-rate domain (nano-$\dot{\varepsilon }M$) experiments, a storage modulus master curve was extrapolated by fitting experimental *E*′(*f*) data from DMA to the following sigmoidal function of $\mathrm{ln}(\omega )$ (Eq. 10)^11^:

10 $$E^{\prime} (\omega )=a\cdot \,\tanh (b\cdot (\mathrm{ln}(\omega )+c))+d$$

where *ω* = 2*πf* represent the angular frequency, while *a*, *b*, *c* and *d* represent the fit coefficients. This sigmoidal fitting function assumes that the mechanics of the tested material can be described by a simple rheological behaviour with one characteristic relaxation frequency (or time) - a good assumption for PDMS^33,38–40^. In particular, the storage modulus master curve presents only one smooth step transition, corresponding to one peak in the loss modulus frequency spectrum, and the behaviour is asymptotic when going to either zero or infinity frequency. This function satisfies the physical requirement of positive and bounded behaviour of the relaxation function at both zero and infinity frequency if *d* > *a*^35^. In case of multiple *E*′ smooth transitions (and concomitant *E*″ peaks), a combination of sigmoidal functions like Eq. 10 is needed to describe the experimental behaviour and yield the entire relaxation function^11,36^, while keeping the analysis steps below unaltered.

The frequency-domain storage modulus function obtained from the fitting, *E*′(*ω*), was then converted into its respective time-domain relaxation modulus function, *E*(*t*), by solving numerically the following integral from the linear theory of viscoelasticity^11,35,36^

11 $$E(t)=\frac{2}{\pi }{\int }_{0}^{\infty }\,\frac{E^{\prime} (\omega )}{\omega }sin(\omega t)\,\partial \omega $$

The stress-time response to a given strain history can be derived from the time-domain relaxation function according to the following convolution integral^35^:

12 $$\sigma (t)=E\,\ast \,\partial \varepsilon ={\int }_{-\infty }^{t}\,E(t-\tau )\frac{\partial \varepsilon (\tau )}{\partial \tau }\partial \tau $$

where *σ* denotes the stress, *ε* the strain, and *τ* a time variable used for integration.

For a constant strain-rate deformation input $\dot{\varepsilon }$ beginning at *t* = 0, the convolution integral in Equation 12 simplifies to:

13 $$\sigma (t)=\dot{\varepsilon }{\int }_{0}^{t}\,E(\tau )\partial \tau $$

The stress-strain response was then obtained by numerical integration of *σ*(*t*) from Equation 13, followed by a linear transformation of the independent variable time (*t*) into strain ($\varepsilon =t\cdot \dot{\varepsilon }$).

Stress-strain responses were computed for each of the 5 strain-rates investigated with the nano-$\dot{\varepsilon }M$ and then used to calculate DMA-derived strain-rate dependent apparent elastic moduli, ${E}_{app,DMA}(\dot{\varepsilon })$, as stress-strain slope within the same LVR used for ${E}_{app,P}(\dot{\varepsilon })$ values from nano-$\dot{\varepsilon }M$ measurements. Thanks to this procedure, ${E}_{app,DMA}(\dot{\varepsilon })$ and ${E}_{app,P}(\dot{\varepsilon })$ data can be directly and meaningfully compared. Calculations to obtain strain-rate dependent stress-strain responses from DMA frequency-dependent data were performed in Mathematica (Wolfram Research Inc., IL, USA).

## Data Availability

All raw and processed data that support the findings of this study are available from the corresponding author upon reasonable request.

## References

[1] Oyen ML (2013). Nanoindentation of biological and biomimetic materials. Exp. Tech..

[2] Oliver WC, Pharr GM (1992). An improved technique for determining hardness and elastic modulus using load and displacement sensing indentation experiments. J. Mater. Res..

[3] Bhushan B, Koinkar VN (1994). Nanoindentation hardness measurements using atomic force microscopy. Appl. Phys. Lett..

[4] Ebenstein DM, Pruitt LA (2006). Nanoindentation of biological materials. Nano Today.

[5] Ebenstein, D. M. Nanoindentation of soft tissues and other biological materials. *Handb. Nanoindentation with Biol*. …, 10.1201/b12116-10 (2010).

[6] Farine, M. Instrumented Indentation of Soft Materials and Biological Tissues. (2013).

[7] Mattei G, Cacopardo L, Ahluwalia A (2017). Micro-Mechanical Viscoelastic Properties of Crosslinked Hydrogels Using the Nano-Epsilon Dot Method. Materials (Basel)..

[8] Bartolini L (2017). Multimodal probe for optical coherence tomography epidetection and micron-scale indentation. J. Innov. Opt. Health Sci..

[9] Mattei G, Ahluwalia A (2016). Sample, testing and analysis variables affecting liver mechanical properties: A review. Acta Biomater..

[10] van Hoorn H, Kurniawan NA, Koenderink GH, Iannuzzi D (2016). Local dynamic mechanical analysis for heterogeneous soft matter using ferrule-top indentation. Soft Matter.

[11] Zeltmann SE, Bharath Kumar BR, Doddamani M, Gupta N (2016). Prediction of strain rate sensitivity of high density polyethylene using integral transform of dynamic mechanical analysis data. Polymer (Guildf)..

[12] Jakes JE, Stone DS (2011). The edge effect in nanoindentation. Philos. Mag..

[13] Shen L, Liu T, Lv P (2005). Polishing effect on nanoindentation behavior of nylon 66 and its nanocomposites. Polym. Test..

[14] Jiang WG, Su JJ, Feng XQ (2008). Effect of surface roughness on nanoindentation test of thin films. Eng. Fract. Mech..

[15] Constantinides G, Ravi Chandran KS, Ulm F-J, Van Vliet KJ (2006). Grid indentation analysis of composite microstructure and mechanics: Principles and validation. Mater. Sci. Eng. A.

[16] Kaufman JD, Miller GJ, Morgan EF, Klapperich CM (2008). Time-dependent mechanical characterization of poly(2-hydroxyethyl methacrylate) hydrogels using nanoindentation and unconfined compression. J. Mater. Res..

[17] Wrucke AJ, Han CS, Majumdar P (2013). Indentation size effect of multiple orders of magnitude in polydimethylsiloxane. J. Appl. Polym. Sci..

[18] Cohen SR, Kalfon-Cohen E (2013). Dynamic nanoindentation by instrumented nanoindentation and force microscopy: A comparative review. Beilstein Journal of Nanotechnology.

[19] Mattei G, Tirella A, Gallone G, Ahluwalia A (2014). Viscoelastic characterisation of pig liver in unconfined compression. J. Biomech..

[20] Tweedie CA, Van Vliet KJ (2006). Contact creep compliance of viscoelastic materials via nanoindentation. J. Mater. Res..

[21] Herbert EG, Oliver WC, Pharr GM (2008). Nanoindentation and the dynamic characterization of viscoelastic solids. J. Phys. D. Appl. Phys..

[22] Kim Y-C (2018). Indentation size effect for spherical nanoindentation on nanoporous gold. Scr. Mater..

[23] Voyiadjis GZ, Peters R (2010). Size effects in nanoindentation: an experimental and analytical study. Acta Mech..

[24] F. Fernando, G. *Introduction to polymer viscoelasticity*. *Materials Characterization* **59** (2008).

[25] Shaw, M. T. & MacKnight, W. J. *Introduction to Polymer Viscoelasticity: Third Edition*, 10.1002/0471741833 (2005).

[26] Heymans N (2003). Constitutive equations for polymer viscoelasticity derived from hierarchical models in cases of failure of time-temperature superposition. Signal Processing.

[27] Maugis D (1992). Adhesion of spheres: The JKR-DMT transition using a dugdale model. J. Colloid Interface Sci..

[28] Zhao YP, Shi X, Li WJ (2003). Effect of work of adhesion on nanoindentation. Rev. Adv. Mater. Sci..

[29] Han L (2011). Time-Dependent Nanomechanics of Cartilage. Biophys. J..

[30] Mencik J, He LH, Swain MV (2009). Determination of viscoelastic-plastic material parameters of biomaterials by instrumented indentation. J. Mech. Behav. Biomed. Mater..

[31] Stroiny A, Gerberich WW (1998). Experimental Analysis of Viscoelastic Behavior in Nanoindentation. Mater. Res. Soc. Symp. Proc..

[32] Chavan, D. *et al*. Ferrule-top nanoindenter: An optomechanical fiber sensor for nanoindentation. *Rev. Sci. Instrum*. **83** (2012). 10.1063/1.4766959 23206101

[33] Mattei G, Gruca G, Rijnveld N, Ahluwalia A (2015). The nano-epsilon dot method for strain rate viscoelastic characterisation of soft biomaterials by spherical nano-indentation. J. Mech. Behav. Biomed. Mater..

[34] Christensen RM (1972). Restrictions Upon Viscoelastic Relaxation Functions and Complex Moduli. Trans. Soc. Rheol..

[35] Christensen, R. M. *Theory of viscoelasticity: an introduction* (Academic Press, 1982).

[36] Zeltmann SE, Prakash KA, Doddamani M, Gupta N (2017). Prediction of modulus at various strain rates from dynamic mechanical analysis data for polymer matrix composites. Compos. Part B Eng..

[37] Hisyam A. Razak A, Szabo P, Skov AL (2015). Enhancement of dielectric permittivity by incorporating PDMS-PEG multiblock copolymers in silicone elastomers. RSC Adv..

[38] Niu T, Cao G, Xiong C (2016). Fracture behavior of graphene mounted on stretchable substrate. Carbon N. Y..

[39] Jiang D, Feng X, Qu B, Wang Y, Fang D (2012). Rate-dependent interaction between thin films and interfaces during micro/nanoscale transfer printing. Soft Matter.

[40] Tirella A, Mattei G, Ahluwalia A (2014). Strain rate viscoelastic analysis of soft and highly hydrated biomaterials. J. Biomed. Mater. Res. Part A.

[41] Charitidis CA (2011). Nanoscale Deformation and Nanomechanical Properties of Polydimethylsiloxane (PDMS). Ind. Eng. Chem. Res..

[42] Kaufman JD, Klapperich CM (2009). Surface detection errors cause overestimation of the modulus in nanoindentation on soft materials. J. Mech. Behav. Biomed. Mater..

[43] Khanafer K, Duprey A, Schlicht M, Berguer R (2009). Effects of strain rate, mixing ratio, and stress–strain definition on the mechanical behavior of the polydimethylsiloxane (PDMS) material as related to its biological applications. Biomed. Microdevices.

[44] Lakes, R. S. *Viscoelastic materials*. (Cambridge University Press, 2009).

[45] Mattei, G. & Ahluwalia, A. A new analytical method for estimating lumped parameter constants of linear viscoelastic models from strain rate tests. *Mech. Time-Dependent Mater*, 10.1007/s11043-018-9385-0 (2018).

[46] Öchsner, A. & Altenbach, H. *Mechanical and Materials Engineering of Modern Structure and Component Design*. **70** (Springer International Publishing, 2015).

[47] Jelen C (2013). Bone scaffolds with homogeneous and discrete gradient mechanical properties. Mater. Sci. Eng. C.

[48] Mattei G (2017). On the adhesion-cohesion balance and oxygen consumption characteristics of liver organoids. PLoS One.

[49] Tirella A (2015). Nano-in-Micro Self-Reporting Hydrogel Constructs. J. Biomed. Nanotechnol..

[50] Mammoto T, Mammoto A, Ingber DE (2013). Mechanobiology and developmental control. Annu. Rev. Cell Dev. Biol..

[51] Mattei G, Ferretti C, Tirella A, Ahluwalia A, Mattioli-Belmonte M (2015). Decoupling the role of stiffness from other hydroxyapatite signalling cues in periosteal derived stem cell differentiation. Sci. Rep..

[52] Cappella, B. *Mechanical Properties of Polymers Measured through AFM Force-Distance Curve*s, 10.1007/978-3-319-29459-9 (Springer International Publishing, 2016).

[53] Mark JE (1999). Polymer data handbook. J. Am. Chem. Soc..

[54] Beekmans SV, Iannuzzi D (2015). A metrological approach for the calibration of force transducers with interferometric readout. Surf. Topogr. Metrol. Prop..

[55] Beekmans SV, Emanuel KS, Smit TH, Iannuzzi D (2017). Minimally Invasive Micro-Indentation: mapping tissue mechanics at the tip of an 18G needle. Sci. Rep..

[56] Roylance D (2001). Engineering viscoelasticity. Dep. Mater. Sci. Eng. Inst. Technol. Cambridge MA.

